# NDIR Gas Sensor for Spatial Monitoring of Carbon Dioxide Concentrations in Naturally Ventilated Livestock Buildings

**DOI:** 10.3390/s150511239

**Published:** 2015-05-13

**Authors:** Luciano B. Mendes, Nico W. M. Ogink, Nadège Edouard, Hendrik Jan C. van Dooren, Ilda de Fátima F. Tinôco, Julio Mosquera

**Affiliations:** 1 Institute for Agricultural and Fisheries Research (ILVO), Technology and Food Science Unit (T&V), Burg. Van Gansberghelaan 115, 9820 Merelbeke, Belgium; 2 Wageningen UR Livestock Research, P.O. Box 135, 6700 AC Wageningen, The Netherlands; E-Mails: nico.ogink@wur.nl (N.W.M.O.); hendrikjan.vandooren@wur.nl (H.J.C.D.), julio.mosquera@wur.nl (J.M.); 3 Department of Agricultural Engineering, Federal University of Viçosa, Av. P. H. Rolfs, S/N, Viçosa, 36570-000, Minas Gerais, Brazil; E-Mail: iftinoco@ufv.br; 4 4INRA-AgrocampusOuest, UMR1348 PEGASE, Domaine de la Prise, 35590 Saint Gilles, France; E-Mail: nadege.edouard@rennes.inra.fr

**Keywords:** ventilation rate, CO_2_ mass balance, dairy barn, open-path laser, photo acoustic spectroscopy

## Abstract

The tracer gas ratio method, using CO_2_ as natural tracer, has been suggested as a pragmatic option to measure emissions from naturally ventilated (NV) barns without the need to directly estimate the ventilation rate. The aim of this research was to assess the performance of a low-cost Non-Dispersive Infra-Red (NDIR) sensor for intensive spatial field monitoring of CO_2_ concentrations in a NV dairy cow house. This was achieved by comparing NDIR sensors with two commonly applied methods, a Photo-Acoustic Spectroscope (PAS) Gas Monitor and an Open-Path laser (OP-laser). First, calibrations for the NDIR sensors were obtained in the laboratory. Then, the NDIR sensors were placed in a dairy cow barn for comparison with the PAS and OP-laser methods. The main conclusions were: (a) in order to represent the overall barn CO_2_ concentration of the dairy cow barn, the number of NDIR sensors to be accounted for average concentration calculation was dependent on barn length and on barn area occupation; and (b) the NDIR CO_2_ sensors are suitable for multi-point monitoring of CO_2_ concentrations in NV livestock barns, being a feasible alternative for the PAS and the OP-laser methods to monitor single-point or averaged spatial CO_2_ concentrations in livestock barns.

## 1. Introduction

Nowadays, no reference method exists for measuring air ventilation rates in naturally ventilated (NV) animal houses. However, a number of different candidate approaches have been suggested [1], such as the tracer gas technique using either natural [2,3,4,5,6,7,8] or artificial tracers [9,10,11,12]. The application of carbon dioxide (CO_2_) as a tracer gas for measuring ventilation and emission rates in livestock buildings involves CO_2_ metabolically produced by the animals and manure, which presents good mixing with most of the target pollutant gases found in livestock houses [13]. 

Representative sampling of the pollutant-tracer ratio is the most robust approach for quantifying emissions [1], and in case of inappropriate mixing spatial variability of this ratio should be included in the sampling strategy. The CO_2_ mass balance relies: (a) on accurate measurements of CO_2_ concentration in- and outside the animal barn; (b) on accurate prediction of the metabolic heat production; and (c) accurate registration of the parameters used in the heat and CO_2_ production model [13,14]. Measuring gaseous concentration distribution in NV livestock structures represents a real challenge in research [15]. For instance, Lefcourt, [16] showed that incorrect selection of sampling positions for ammonia (NH_3_) in NV animal barns may lead to errors in calculated NH_3_ emission rates from 50% to over 200% of the actual values. It is widely recognized that the best position to achieve a representative average gas concentration is at the air outlets of the building; however, in NV buildings inlet and outlet positions are critically dependent on meteorological conditions and local topography, and therefore, the proper selection of inlets and outlets is not trivial [1]. The situation becomes even more complex in very open livestock housing structures, where due to the expected high spatial and temporal variability, use of a measurement system with high spatial and temporal resolution may be required. One possible solution for this issue is by multi-point monitoring CO_2_ concentrations in a naturally ventilated livestock barn, and determination of spatial concentration profiles. Furthermore, currently used measurement instruments to monitor CO_2_ concentrations are often expensive, or present limitations when used to sequentially measure points, resulting in complex, costly and labor intensive systems [17]. 

Currently applied systems to monitor CO_2_ concentrations from agricultural facilities include Photo Acoustic Spectroscope (PAS) Gas Analyzer [4,6,18,19,20,21,22,23,24,25]; and the Open-Path laser (OP-laser) [26,27,28,29,30]. The OP-laser and PAS analyzer have been extensively used by researchers seeking to monitor emissions from livestock barns in the last years, mostly mechanically ventilated. However, when it comes to monitoring gaseous concentrations in naturally ventilated livestock barns, the spatial variability in concentration profiles becomes an important issue and measuring concentrations in many points turns crucial. An important disadvantage of applying either PAS or OP-laser is the high purchase cost, in particular when multiple sampling is required. Due to the large spatial variability in livestock buildings, the use of a cheaper CO_2_ monitoring system, allowing for multi-point sampling, may result in similar levels of accuracy than using more accurate (and more expensive) methods. 

In this paper, Non-Dispersive Infra-Red (NDIR) based sensors are suggested as an alternative for PAS and OP-laser to measure CO_2_ concentrations in NV buildings. In the studies of Piccot *et al*. [31] and Yasuda *et al*. [32], several different commercially available NDIR CO_2_ sensors were compared with a reference method in the laboratory, and concluded that concentrations measured with all sensors agreed well with that of the calibration gas. However, field performance of the selected NDIR technique is still meager in current literature.

The goal of this study was to assess the performance of a low-cost NDIR sensor for intensive spatial field monitoring of CO_2_ concentrations in a livestock building, as compared to the OP-laser and PAS methods. Specific objectives of this research were: (a) to test the NDIR sensor in the laboratory for linearity, variability between sensors and sensitivity to ambient static pressure and (b) to compare measurements from NDIR sensors with OP-laser and PAS methods in a NV dairy cow barn.

## 2. Material and Methods

In order to assess their feasibility for use in NV livestock barns, the NDIR sensors were first tested in the laboratory for linearity, variability between sensors and sensitivity to static pressure. Subsequently, the sensors were brought to the field for exposure to actual CO_2_ concentrations in a NV dairy cow barn, and compared with two other measuring devices: the PAS analyzer and the OP-laser. Bland-Altman charts were plotted to assess the agreement between the compared measurement techniques. A detailed description of the methods, experimental procedures and data analysis conducted to meet the objectives of this study is provided below.

### 2.1. Description of the Carbon Dioxide Measuring Devices

#### 2.1.1. NDIR CO_2_ Gas Sensor 

**Figure 1:**
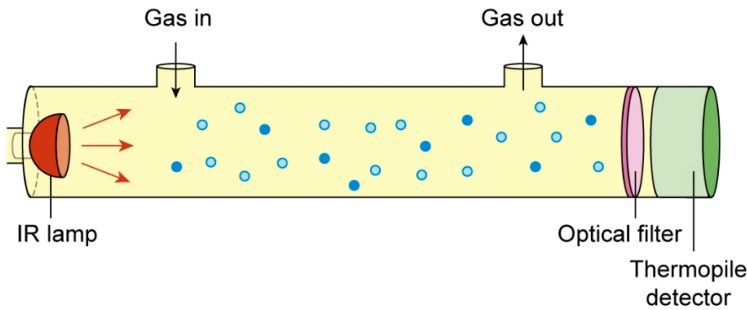
Sketch of the Non Dispersive Infra-Red (NDIR) carbon dioxide (CO_2_) sensor structure.

It is a portable sensor (model SD-GAS-025, Sensor Data B. V., Rijswijk, The Netherlands), with measuring principle based on gas absorption of radiation at a known wavelength [33,34,35]. Its core part consists (Figure 1) of a NDIR module which is comprised of an infrared source, a measuring chamber, a wavelength filter and an infrared detector (CO_2_ Engine^®^ K30, Delsbo, Sweden). Any CO_2_ molecules present inside of the measuring chamber will only absorb a specific wavelength of the light given off by the infrared source. The filter allows only the particular wavelength of 4.3 µm to pass through it. The light intensity that is received by the detector is then proportional to the number of given CO_2_ molecules inside the chamber and can be described through the Lambert-Beer’s Law in Equation (1):

I = I_0_ × e^−k×l×[CO_2_]^ (1)

where:

I = light power intensity after absorption by CO_2_, measured at the detector (W·m^−2^);
I_0_ = light power intensity at the source (W·m^−2^);
k = absorption index of CO_2_ at 4.3 μm (dimensionless);
l = length of the absorption path (cm);
[CO_2_] = CO_2_ concentration to be measured (mol·dm^−3^).

The NDIR sensors were connected to a datalogger system (CR1000, Campbell Scientific, Inc., Logan, UT, USA) located in a shelter placed outside the barn, each sensor was powered with a voltage of 1200 mV. For the laboratory comparisons, average concentrations of CO_2_ concentrations over periods of 5 min was used, while during field tests, CO_2_ concentrations were integrated over a 60 min period. 

#### 2.1.2. OP-Laser

**Figure 2:**
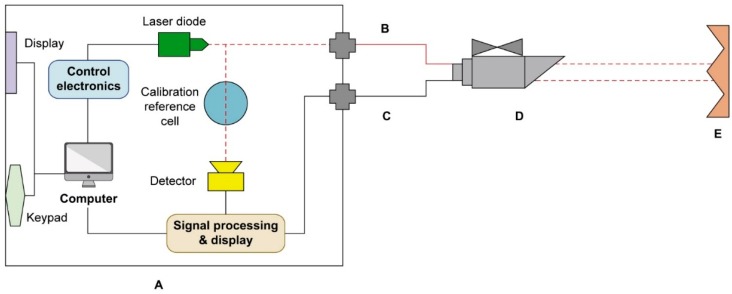
Schematic representation of the OP-laser components. (**A**) open-path gas monitor; (**B**) fiber-optical cable; (**C**) co-axial cable; (**D**) remote head; and (**E**) retro-reflector.

This device (model GasFinderFC, Boreal Laser, Alberta, Canada) measures the average gaseous CO_2_ concentration in the air through a user set open path length. The OP-laser is composed of a single channel gas monitor, a fiber-optical cable (type SMF-28, mode diameter of 9 μm, single mode with FC/APC, 3 m long), a co-axial cable (Belden 9880, 3 m long), a remote head (model OP3) and a retro-reflector (Figure 2). The OP-laser was set up to measure CO_2_ concentrations in the central axis or the barn (path length of 64 m), at approximately the same level as the NDIR sensors (3 m above the slatted floor). The remote retro-reflector (prism like mirror), made of aluminum and coated with a thin layer of gold (to ensure high reflectance and no tarnishing) was installed on the wall at the opposite side of the barn for reflection of the laser beam back to the source. The retro-reflector mirror was housed inside a sealed enclosure made out of polyethylene with a polycarbonate window.

The used OP-laser was sent to factory for maintenance within 12 months prior to the execution of the experiments. It holds a self-calibration check mechanism, which includes a reference calibration cell (crystal sphere containing CO_2_ at a known concentration, Figure 2); a portion of the laser beam generated by the laser diode is passed through the reference cell every minute to provide continuous update on calibration quality. The waveform of each path is first processed and then compared to determine the adsorption in the measurement path. The reference cell of known concentration is used to determine the adsorption of the other path. These curves (the standard reference and the sample waveforms) are then digitized and compared as two numeric arrays through the linear least squares regression analysis, from which a coefficient of determination (R^2^) and the slope of the regression line are obtained. Any significant deviation generates a status code which alerts the user to a potential calibration problem. 

**Figure 3:**
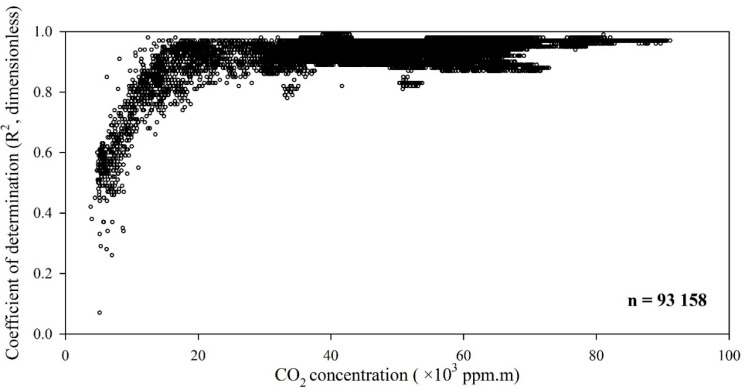
Improvement of the coefficient of determination (R^2^) with increase in CO_2_ concentration (ppm∙m) measured at the dairy cow house with the OP-laser (path length 64 m).

The CO_2_ concentration measured in the dairy cow house with the OP-laser was pre-analyzed and the plot of concentration *versus* the R^2^ was made and presented in Figure 3. The plot was used to determine the sensitivity of the OP-laser to the gas concentration measurement at field conditions and a minimum threshold of R^2^ = 0.80, as a measure for minimum measurement quality was applied to filter CO_2_ concentration data in this study. 

#### 2.1.3. PAS Analyzer 

**Figure 4:**
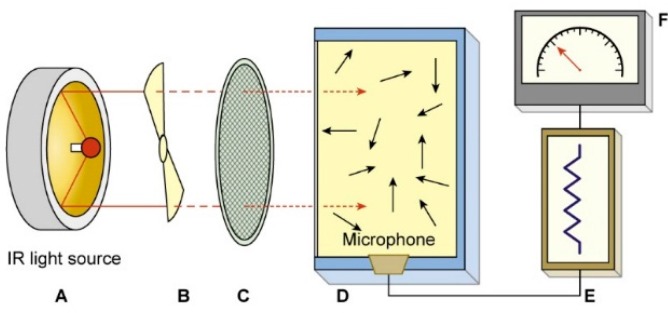
Schematic representation of the main components of a photoacoustic spectroscopy (PAS) analyser. (**A**) Reflector; (**B**) chopper; (**C**) filter; (**D**) photoacoustic chamber; (**E**) signal processor and (**F**) output display.

It is based on the photo-acoustic principle, where chopped IR light is converted into an acoustic signal measured by a highly sensitive microphone placed on the wall of the measurement chamber. Inside the PAS analyzer (model 1312, INNOVA AirTech Instruments A/S, Ballerup, Denmark), a sample of air is sealed in the measurement chamber; then, the chamber is irradiated with pulsed narrow-band IR light. The light absorbed by the gas sample present in the chamber, which is proportional to the gaseous concentration, is converted into heat. The light chopper causes the air sample to heat and cool down, while the temperature fluctuations generate a pressure wave signal, which is detected by the microphone, which is subsequently digitized and converted into gaseous concentration (Figure 4). 

The PAS analyzers used in this study were set to measure CO_2_ with IR light filter (model UA0982, Air Tech Instruments), which only let pass through IR light with a center wave length of 14.1 µm ± 7.5%. The monitor was set to sample at an interval of 40 s plus 10 s for flushing, and a time integration interval of 5 min. Dew point temperature (T_dp_) was also monitored by the PAS in order to account for cross-interference with moisture in the air. The PAS analyzers were sent to the manufacturer for certified calibration against CO_2_ and other gases along with crossed interference with water vapor within 6 to 12 months prior to the beginning of the trials, in order to make sure that deviation on concentration measurements was under the tolerance level of 5%. Some additional technical specifications of all CO_2_ measuring devices used in this study are presented in Table 1.

**Table 1:** Technical specifications of the CO_2_ measuring devices used in this study.

	NDIR Sensor	OP-Laser	PAS Analyzer
**Operating principle**	Non-Dispersive Infra-Red	Tunable Diode Laser Absorption Spectroscopy	Photoacoustic spectroscope
**Target gas**	CO_2_ only	CO_2_ only	CO_2_ and others
**Tuned wavelength for detection of CO_2_ (µm)**	4.3	1.6	14.1
**Detection limit**	0.2 ppm_v_	2000 ppm∙m	1.5 ppm_v_
**Accuracy**	±30 ppm_v_ ± 2%	500 ppm∙m	±1%
**Response time (T_1/e_)**	20 s (diffusion time)	1 s (scan rate)	25 s (for 1 gas, including flushing period and 5 s sampling integration time)
**Operating temperature (°C)**	0 to +50 °C	−45 °C to +80 °C	−20 °C to +70 °C
**Sensitivity to pressure ^1^**	0.1%	Not determined	0.5%
**Type of calibration**	Two-points calibration in the laboratory	Continuous self-calibration mechanism with a reference gas crystal cell	Two-points calibration at factory
**Laboratory check of calibration with certified gas**	Yes	Yes	Yes
**Cross-compensation for water vapor**	Not needed	Not needed	Yes
**Cost per unit (€) ^2^**	300	35,000	28,000

^1^ Specified by factory; ^2^ Approximate, excluding costs with external datalogger systems, when applicable.

### 2.2. Laboratory Tests and Calibration of NDIR Sensors

Prior to their installation in the animal house, the NDIR sensors were tested in the laboratory for output linearity and sensitivity to static pressure. For the test of linearity, span gas at 1952 ppm_v_ concentration of CO_2_ (certified concentration accuracy of ±20 ppm_v_) was diluted with zero gas (N_2_, certified purity of 99.999%) at the percentages of 0%, 10%, 20%, 30%, 40%, 50%, 60%, 70%, 80%, 90% and 100% of the span gas, resulting in concentrations of 1952, 1757, 1562, 1366, 1171, 976, 781, 586, 390, 195 and 0 ppm_v_ of CO_2_, respectively. The dilution was achieved with the aid of a precision gas divider (model 821, Signal Instruments Co., Camberley, UK), which operates by dividing the flow rates of the incoming gases down a chain of ten identical capillary tubes, in such a way that the dilution level will be the ratio between capillaries carrying span and zero gases. To avoid contamination of the gas mixture with other gases, after being produced by the gas divider, the mixture was immediately directed into the NDIR during the test of linearity through polytetrafluoroethylene (PTFF) tubing. One NDIR sensor was exposed to gas at each concentration, at a flow rate of 200 mL∙min^−1^. This flow rate value was used to apply into sensors an average static pressure to which they were usually exposed during field measurements. After stabilization of the sensor output (mV), a few readings were recorded. During the laboratory exposure trials, gaseous mixture static pressure and temperature at the entrance to the sensor remained approximately constant and equal to (1006 ± 1) hPa and (24 ± 1) °C, respectively.

For the static pressure sensitivity test, CO_2_ span gas at 1952 ppm_v_ was passed through a NDIR sensor at the following relative pressures: 1.5, 4.5, 9.0, 15.0, 23.0 and 33.0 hPa. The relative pressure values (relative to room static pressure) were achieved in the laboratory by applying an overpressure inside a sealed protecting case where the sensor was housed. After full reading stabilization, sensor raw data was recorded for each static pressure level. Air temperature was kept approximately constant and equal to (24 ± 1) °C during the test. The sensitivity of the NDIR sensors to static pressure was calculated according to Equation (2):

S_sp_ = ((Δ_raw_)/(Avg_raw_×Δ_sp_)) × 100 (2)

where:

S_sp_ = sensitivity of the NDIR to change in static pressure (% of change in raw data per unit of pressure);
Δ_raw_ = measured range of raw data (mV);
Avg_raw_ = average raw data value (mV);
Δ_sp_ = measured range of pressure (hPa).

To account for sensor individual variability and to allow independent direct comparison with the reference calibration gases, a two-point calibration was developed in the laboratory for every NDIR sensor. Sensors were excited with a voltage of 1200 mV and exposed to calibration gases at 0 ppm_v_ of CO_2_ (N_2_, certified purity of 99.999%) and 1952 ppm_v_ (span, certified accuracy of ±20 ppm_v_) at a flow rate of 200 mL∙min^−1^. During exposure to each gas, sensors that presented drifted readings were adjusted. Sensor raw data was corrected for the reference static pressure of 1013.25 mbar and then correlated to the reference gas concentrations. This procedure was performed twice for every sensor: before the start of the field measurements, and at the end of the trials. The final calibration equation was obtained by using data from both calibration procedures, resulting in a single equation per sensor.

The response time of the NDIR sensors to span gas concentrations at the flow rate of 200 mL·min^‒1^ took 15 to 30 s and was considered relatively small compared to the data retrieval rate used in practice of 5 min.

### 2.3. Description of the Livestock Barn

**Figure 5:**
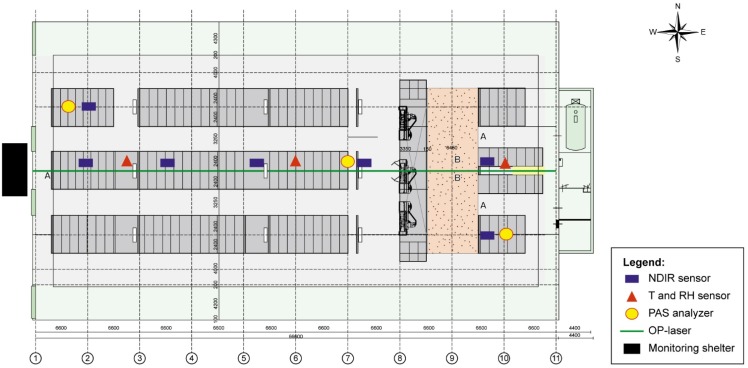
Plan view of the investigated barn with the allocation of the different carbon dioxide (CO_2_) measuring devices and temperature (T) and relative humidity (RH) sensors (not to scale). The approximate distance between NDIR sensors in the central axis is 13 m.

This study took place in a dairy cow barn (Figure 5). The barn is located in the county Bunschoten, in the middle of the Netherlands, is east-west oriented, has a roof with 37% slope, and has dimensions of 64 m Length × 38 m Width × 4 m Heigth (L × W × H). The building envelope is composed of insulated roof and side walls, the lateral openings on both sides are 2.75 m high, protected with stainless steel screens with openings of 5 × 5 cm^2^ and has manually operated curtains. In the eastern part of the building there is a deep litter area of 10 m L × 21 m W with maximum housing capacity of 30 dry and pregnant cows. In the central part of the building, 3 double-rows of cubicles (paper chips bedding; 42 m L × 21 m W) are located, with feeding alleys on both sides (north and south), and maximum housing capacity for 150 lactating cows. The last section of the barn is at the most western side, has an area of 13 m L × 21 m W with similar cubicles and bedding system as for the lactating cows, where the heifers are kept (maximum capacity of 40 heifers). Barn cubicles area had slatted walking alleys and an automatic scraping robot. Manure is stored in a deep pit space of 65 m L × 21 m W × 2 m Depth located under the slats and cubicles. Manure was removed from the barn twice a year, usually during early spring and fall seasons. The lactating cows had free access to three milking robot systems. All cows were kept inside year round and were fed with roughage (grass and corn silage) and concentrate. The data collection and validation trials in the barn were conducted during the summer season, in the months of June, July and August of 2012. A more detailed description of the experimental barn is provided by Mendes *et al*. [36].

### 2.4. Field Experimental Setup and Data Collection Procedure

Prior to each measurement campaign, the OP-laser was installed in the barn to sample the average CO_2_ concentration in an open-path of 64 m along the central axis of the barn (3 m from the slats, 19 m of distance from each side). For the comparison of NDIR sensors with the OP-laser, five sensors were placed in the central axis of the barn (Figure 5). The central line of NDIR sensors was located at a height of 3 m above the slats, 19 m from both side walls. The distance between sensors being approximately 13 m, where the first and last sensors were 6 m away from front and back walls. Measurements with both NDIR sensors and OP-laser were taken for a minimum of 48 h per trial, with a total of six trials. To reduce the effect of sensor individual variability, five NDIR sensors were, in every trial, randomly selected from a set of 17 new sensors. Data sets from all trials were pooled together and hourly averaged data points were calculated prior to statistical analyses.

For the comparison of the NDIR sensors with the PAS analyzer, three air sampling ports were installed to collect samples from three fixed different points inside the barn (Figure 5). The PAS analyzers were placed inside the shelter located at the west part outside the building. The sampling lines consisted of PTFF tubes (0.63 cm internal diameter) with PTFF filters (4.7 cm diameter, 5 μm pore diameter) installed at the end. The air was drawn by the internal pump of the PAS analyzers, with a flow rate of 0.108 m^3^∙h^−1^ during measurements and 0.018 m^3^∙h^−1^ when flushing the measurement chamber. Each PAS analyzer was programmed to sample CO_2_ concentrations at every 40 s, with 10 s for flushing, and to store average values at every 5 min. Data was downloaded from the analyzers at the end of each measurement. During every trial, three NDIR sensors were installed at the same height and next to each of the PAS analyzers’ sampling ports (within a maximum distance of 0.5 m) for simultaneous measurement of CO_2_ concentration. A total of three trials were performed, each lasting a minimum of 48 h. During each trial, the three used NDIR sensors were randomly selected from a pool of 17 new sensors, in order to take into account the effect of sensor individual variability. Data sets for all performed trials were pooled together prior to the statistical analyses, and hourly averaged values were calculated for each measuring device, for posterior analysis.

### 2.5. Analysis of Agreement between Laboratory and Field Comparison Methods

The agreement between CO_2_ concentration data from the NDIR sensors compared to the concentration data from the OP-laser and PAS analyzer was assessed by regressing the difference between CO_2_ concentrations (Diff[CO_2_]) determined with the NDIR method and OP-laser or PAS analyzer and the average CO_2_ concentration (Avg[CO_2_]). Results were compared by using the analysis of agreement of Altman and Bland [37], by regressing Diff[CO_2_] and Avg[CO_2_] according to the linear model in Equation (3):

Diff[CO_2_] = ß_0_ + ß_1_ × Avg [CO_2_] (3)

where:

Diff[CO_2_] = ([CO_2_]_NDIR_ – [CO_2_]_OP-laser or PAS_), difference between CO_2_ concentrations obtained from the NDIR sensors and OP-laser or PAS analyzers, ppm_v_;
β_o_ = Y-intercept, a measure of systematic positive or negative bias, ppm_v_;
β_1_ = Slope, a measure of non-systematic heterogeneous bias, non-dimensional;
Avg[CO_2_] = ([CO_2_]_NDIR_ + [CO_2_]_OP-laser or PAS_)/2; average between concentration measurements obtained from the NDIR sensors and OP-laser or PAS analyzers, ppm_v_.

In Equation (3), the intercept (β_o_) and the slope (β_1_) represent homogeneous and heterogeneous systematic bias, respectively. A test of significance for each coefficient was carried on with *procreg* in SAS^®^ (Version 9.4, Cary, NC, USA) to assess whether β_o_ and β_1_ were statistically different from zero. 

The agreement between NDIR sensors and OP-laser was done by averaging out data from different number of NDIR sensors, *i.e*., 5, 3, 2 and 1 over a time integration interval of 1 h, and comparing with the path averaged concentration measured with the OP-laser. The analysis of agreement between NDIR sensors and PAS analyzers was done using data from both instruments collected at approximately the same point in the barn, also averaging data based on a time integration of 1 h.

## 3. Results and Discussion

### 3.1. Laboratory Tests with the NDIR Sensors

**Figure 6:**
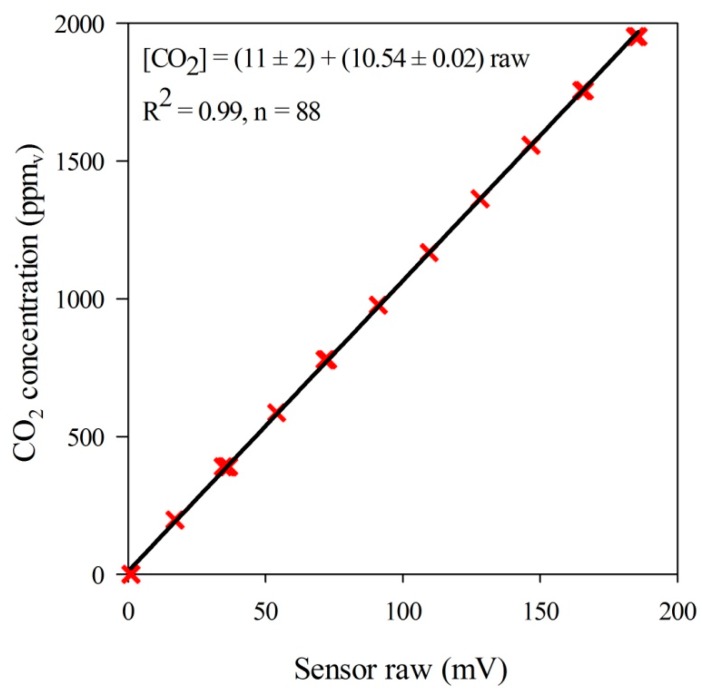
Plot of the linear response of sensor (mV) to calibration gas CO_2_ concentration (ppm_v_).

The results of the linear regression performed for NDIR sensor raw data against CO_2_ concentration is presented in Figure 6.

The performed test of linearity indicates that when CO_2_ concentrations vary in the range of 0 to 1952 ppm_v_, the sensor had a nearly perfect linear response (*p*-value < 0.0001), with intercept and slope of (−11 ± 2) ppm_v_ and (10.54 ± 0.02) ppm_v_∙mV^−1^. Such outcome is in agreement with the linear behavior found by Hodgkinson *et al*. [38] within the same range of CO_2_ concentration levels measured in this study. The analysis of sensitivity to static pressure yielded a S_sp_ value of 0.08% of the reading per each 1 hPa, over the tested range, which agrees to the specifications of the sensor factory of 0.1% [39]. 

Next to the tests of linearity and sensitivity to static pressure conducted with the NDIR sensors, individual calibration was performed in the laboratory and the results are presented in Table 2. When comparing the coefficients of calibration equations within the 17 sensors presented in Table 2, one notices that they present some variability between one another. For instance, when using the sensors to measure a typical CO_2_ concentration of 910 ppm_v_, the range of measurements with the different sensors was within 896 to 942 ppm_v_, resulting in an overall standard error of the mean from all 17 sensors of 4 ppm_v_, which corresponds to a variability between sensors of 5%. 

The developed calibration equations presented in Table 2 were obtained by pooling together data from calibration procedures applied to the sensors twice in a period of three months. During this time frame, the drift in calibration coefficients observed in each individual sensor was estimated to be (0.4 ± 0.4)%, and thus rather negligible, although over longer times of sensor exposure, significant drifts from calibration might occur. However, effects of long exposure time on sensor calibration equations was not the purpose of this study, and might be a subject for further investigation on field application of NDIR sensors. 

**Table 2:** Laboratory two-points individual calibration equations for the NDIR sensors raw data (mV) against CO_2_ concentration ([CO_2_], ppm_v_), for the linear model of the form [CO_2_] = a + b raw.

Sensor ID	a ± SE	b ± SE
03062CE1	−11 ±10	10.40 * ± 0.30
03062CD1	−9 ± 7	10.40 * ± 0.20
03062CDF	−9 ± 8	10.40 * ± 0.20
0306281F	−9 ± 14	10.10 * ± 0.10
0305EFAB	−9 ± 8	10.06 * ± 0.06
0306820	−10 ± 7	10.08 * ± 0.05
0306813	−9.8 * ± 0.5	10.12 * ± 0.03
03062CE0	−10 * ± 1	10.12 * ± 0.01
03062CD5	−10 ± 6	10.13 * ± 0.04
030627C8	−9 ± 7	10.13 * ± 0.05
0305DEF5	−10.4 * ± 0.2	10.13 * ± 0.01
03062CD3	−9.5 * ± 0.4	10.13 * ± 0.03
030627CA	−9.4 * ± 0.2	10.13 * ± 0.01
03062CD4	−10 * ± 3	10.14 * ± 0.02
03062D34	−11 * ± 7	10.61 * ± 0.03
030627CB	−10 * ± 3	10.13 * ± 0.02
03062C0	−15 * ± 6	10.64 * ± 0.01

* Mean values of a and b are significantly different than zero at the level of 95% probability.

### 3.3. Analysis of Agreement of CO_2_ Data from NDIR Sensors against PAS and OP-Laser Data

**Figure 7:**
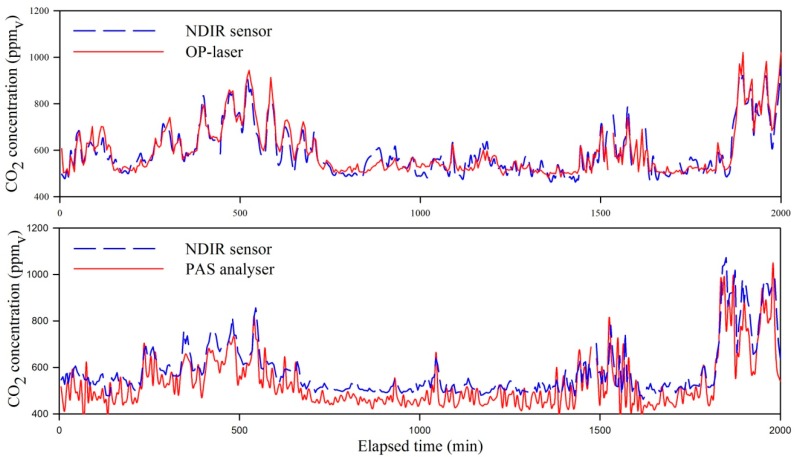
Snapshot of CO_2_ concentrations in the dairy cow barn monitored with NDIR sensors, when compared with the OP-laser while used to measure the mean concentration in an open-path of 64 m in the cow barn with five sensors and the (**top**) and compared with the PAS analyzer on a single-point basis (**bottom**). The top and bottom plots are from data collected in a simultaneous trial.

**Figure 8:**
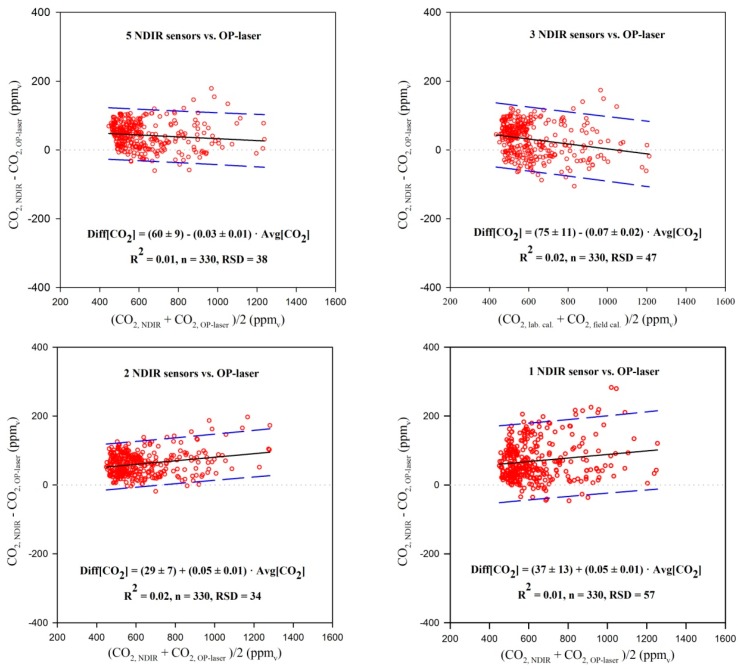
Bland-Altman charts for the comparison of CO_2_ concentrations measured with the NDIR sensors and the OP-laser, averaging out data from different numbers of NDIR sensors. The dashed blue lines represent the 95% confidence interval.

A snapshot of CO_2_ concentration data measured at the dairy cow barn with the NDIR sensors, OP-laser and PAS analyzer is represented graphically in Figure 7. The results of the analysis of agreement between the CO_2_ concentrations measured in the field averaged out from different numbers of NDIR sensors and as compared to the OP-laser are presented in Figure 8. It can be seen that the points presented a more homogeneous distribution around the reference line of Diff[CO_2_] = 0 when data from five NDIR sensors were averaged out, while comparing the OP-laser with only three, two or one NDIR sensors presented a linear trend that drifted from the reference line. This outcome might have stemmed from the fact that fewer than five NDIR sensors will cause the mean CO_2_ concentration value to misrepresent the concentration measured by the OP-laser. From the five NDIR sensors, three were placed in the milking cows area (where most of the metabolic CO_2_ sources are), and the other two were each placed above the dry and young cows’ areas; if less than three NDIR sensors are placed above the milking cows area, the prediction of the CO_2_ concentration from the OP-laser is poorer, as shown by the cases where three, two and one sensors are used. On the other hand, placing CO_2_ sensors above the milking cows area only is not enough to represent barn total CO_2_ concentration, as shown by the cases when data from two or one NDIR sensors in the milking cows area is paired with the data from OP-laser. In these cases, barn CO_2_ concentration is over-estimated as lactating cows produce and emit more CO_2_ than dry or young cows [13].

The 95% level significance test revealed that the term β_o_ in Equation (3) was different from zeroand equaled to (60 ± 9) ppm_v_ when averaging CO_2_ concentration data from 5 NDIR sensors, which was considered relatively small, given the magnitude of practical measurements for dairy cow barns (440 to 2000 ppm_v_). In fact, when applied in practice for CO_2_ mass balance experiments, this bias will be eliminated by using the same sensor for background measurements. A similar outcome was observed by Chagunda *et al*. [40] when comparing a NDIR device to a OP-laser analyzer for measurements of methane (CH_4_) concentrations from cattle. Those authors also observed that the NDIR method systematically overestimated the OP-laser method by 30 ppm_v_, and related the discrepancy to inherent distinct nature of the measurement devices. Chagunda *et al*. [40] added that although different methods resulted in concentrations that were distinct in absolute values, the trends in the measurements were similar.

The estimated value for β_1_ when averaging out data from five NDIR sensors was (−0.03 ± 0.01) ppm_v_∙ppm_v_^−1^, and was not significantly different from zero at the level of 95% confidence, indicating that a systematic proportional underestimation (or heterogeneous bias) of the CO_2_ concentrations measured in the dairy cow barn with five NDIR sensors was not present. 

**Figure 9:**
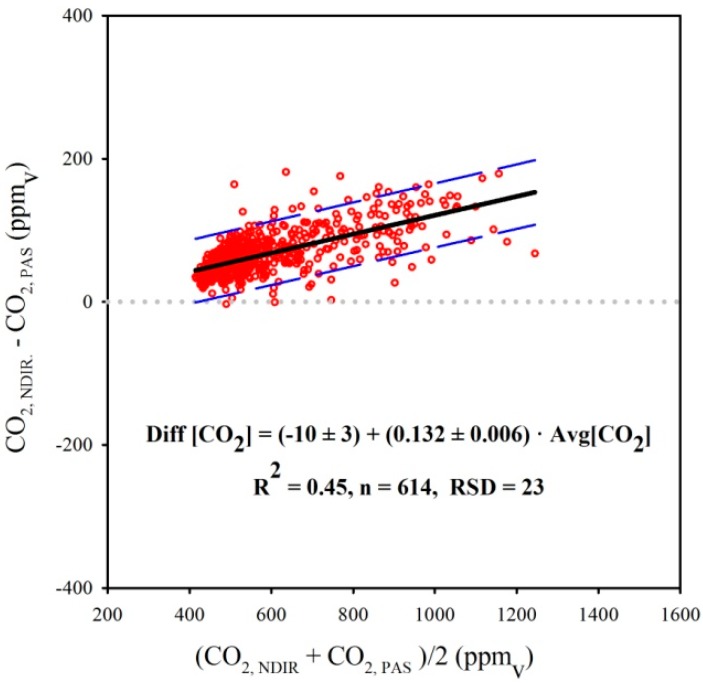
Bland-Altman chart for the relationship of CO_2_ concentration determined with NDIR sensors and PAS analyzers. The dashed blue lines represent the 95% confidence band.

A Bland-Altman plot for CO_2_ concentrations determined with the NDIR sensors against CO_2_ concentrations determined with the PAS analyzers is presented in Figure 9. The majority of the data points are above the line of Diff[CO_2_] = 0, suggesting that the NDIR sensors tended to provide higher CO_2_ concentrations compared to the PAS analyzer. The significance test for β_o_ in Equation (3) yielded to (−10 ± 3) ppm_v_, which is statistically different from zero, but considered negligible in practical situations. 

**Figure 10:**
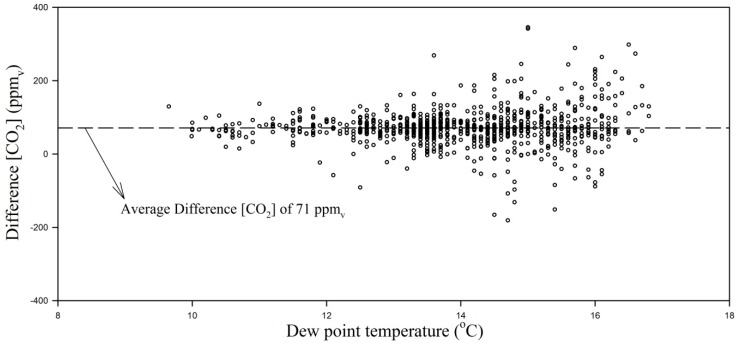
Plot representing the increased absolute difference between PAS and NDIR measurements of CO_2_ concentrations (ppm_v_) in the dairy cow house with increasing dew-point temperature (T_dp_, °C).

Results of the significance test performed on the coefficient β_1_ in Equation (3) demonstrated that a systematic positive heterogeneous bias was present in the measurements of CO_2_ concentrations made with the NDIR sensors in relation to the PAS analyzers (0.132 ± 0.006) ppm_v_∙ppm_v_^−1^, or, 13.2%, and was significantly different from zero (*p*-value < 0.0001). A positive heterogeneous (or proportional) bias indicates that as the mean CO_2_ concentration in the dairy cow house became higher, the difference between concentrations measured with the NDIR sensors and PAS analyzers also enlarged. According to previous research studies, e.g., [23,41,42] PAS analyzers might be affected by cross interference between gaseous CO_2_ and water vapor, especially in livestock buildings where concentrations of CO_2_ and water vapor are usually correlated. Hence, an analysis of cross interference between CO_2_ concentration and water vapor in this study was conducted by plotting a chart with dew-point temperature (T_dp_, °C) *versus* the difference in CO_2_ concentration measured with the NDIR sensors and the PAS analyzer (Figure 10). Results indicate that increasing T_dp_ did not result in higher average values for the discrepancy between CO_2_ concentration measurements from NDIR and PAS; instead, the average discrepancy remained constant (71 ppm_v_), and thus could not be used to explain the positive homogenous systematic error observed in the comparison between PAS and NDIR sensors observed in this study. 

One source of heterogeneous overestimation of NDIR sensors, when compared to the PAS analyzers, might have stemmed from the sampling strategy used to compare both methods, by placing the NDIR sensors from an average distance of 0.5 m from the PAS analyzers. Although every effort was taken in order to make NDIR sensors capture the same concentration as the PAS analyzer, such a task is difficult to achieve given the inherent spatial variability of CO_2_ concentrations present in NV livestock barns, where large concentration gradients may be present even within small distances. The complexity in spatial variability of CO_2_ distribution in NV barns is well known [15], especially when measurements are made at close proximity to the CO_2_ emission sources (the animals), as demonstrated by Mendes *et al*. [36]. In addition, not only CO_2_, but other gases produced in livestock barns present high concentration gradients, such as ammonia (NH_3_) [16,43]. 

## 4. Conclusions

A NDIR sensor technology was tested for monitoring of CO_2_ in a NV dairy cow barn. Laboratory calibration, tests of linearity and sensitivity to static pressure were performed on the sensor. After being calibrated, NDIR sensors were exposed to CO_2_ concentrations in the livestock barn together with PAS analyzers and an OP-laser. An analysis of agreement between the concentrations measured with NDIR sensors and the methods of comparison was performed. The following conclusions can be drawn:

(1) The tested NDIR sensor presents a small variability between sensors of 5%, and a sensitivity to static pressure of 0.08% of the reading per each 1 hPa;
(2) In order to represent the overall CO_2_ concentration of the dairy cow barn, the number of NDIR sensors to be accounted for average concentration calculation is dependent on barn length and on barn area occupation.
(3) The NDIR CO_2_ sensors are suitable for multi-point monitoring of CO_2_ concentrations in NV livestock barns, and is a feasible alternative for the PAS and the OP-laser methods to monitor single-point or averaged spatial CO_2_ concentrations in livestock barns.

## Abbreviations

NV: naturally ventilated
PAS: Photo-Acoustic Spectroscope
NDIR: Non-Dispersive Infra-Red
L: Length dimension
W: Width dimension
H: Height dimension
OP: Open-Path
PTFF: polytetrafluoroethylene

## References

[1] Ogink N.W.M., Mosquera J., Calvet S., Zhang G. (2013). Methods for measuring gas emissions from naturally ventilated livestock buildings: Developments over the last decade and perspectives for improvement. Biosyst. Eng..

[2] Feddes J.J.R., Leonard J.J., McQuitty J.B. (1984). Carbon dioxide concentration as a measure of air exchange in animal housing. Can. Agric. Eng..

[3] Van Ouwerkerk E.N.J., Pedersen S. Application of the carbon dioxide mass balance method to evaluate ventilation rate in livestock buildings. Proceedings of the XII World Congress on Agricultural Engineering.

[4] Heber A.J., Ni J.Q., Haymore B.L., Duggirala R.K., Keener K.M. (2001). Air quality and emission measurement methodology at swine finishing buildings. Trans. Am. Soc. Agric. Eng..

[5] Blanes V., Pedersen S. (2005). Ventilation flow in pig houses measured and calculated by carbon dioxide, moisture and heat balance equations. Biosyst. Eng..

[6] Xin H., Burns R.T., Gates R.S., Overhults D., Earnest J.W. (2009). Use of CO_2_ concentration difference or CO_2_ balance to assess ventilation rate of broiler houses. Trans. ASABE.

[7] Samer M., Berg W., Fiedler M., Von Bobrutzki K., Ammon C., Sanftleben P., Brunsch R. A comparative study among H_2_O-balance, heat balance, CO_2_-balance and radioactive tracer gas technique for airflow rates measurement in naturally ventilated dairy barns. Proceedings of the Ninth International Livestock Environment Symposium (ILES IX).

[8] Mendes L.B., Tinôco I.F.F., Saraz J.A.O., Ogink N.W.M., Xin H. (2014). A refined protocol for calculating ventilation rates of naturally ventilated broiler barns based on metabolic CO_2_ production. DYNA.

[9] Demmers T.G.M., Phillips V.R., Short L.S., Burgess L.R., Hoxey R.P., Wathes C.M. (2001). SE—Structure and Environment: Validation of ventilation rate measurement methods and the ammonia emission from naturally ventilated dairy and beef buildings in the United Kingdom. J. Agric. Eng. Res..

[10] Snell H.G.J., Seipelt F., Weghe H.F.A. (2003). Van den Ventilation rates and gaseous emissions from naturally ventilated dairy houses. Biosyst. Eng..

[11] Wu W., Zhang G., Takai P. (2012). Ammonia and methane emissions from two naturally ventilated dairy cattle buildings and the influence of climatic factors on ammonia emissions. Atmos. Environ..

[12] Kiwan A., Berg W., Fiedler M., Ammon C., Gläser M., Müller H.J., Brunsch R. (2013). Air exchange rate measurements in naturally ventilated dairy buildings using the tracer gas decay method with ^85^Kr, compared to CO_2_ mass balance and discharge coefficient methods. Biosyst. Eng..

[13] Pedersen S., Blanes-Vidal V., Joergensen H., Chwalibog A., Haeussermann A., Heetkamp M.J.W., Aarnink A.J.A. (2008). Carbon dioxide production in animal houses: A literature review. Agric. Eng. Int..

[14] CIGR (2006). Animal Housing in Hot Climates—A multidisciplinary View.

[15] Calvet S., Gates R.S., Zhang G., Estellés F., Ogink N.W.M., Pedersen S., Berckmans D. (2013). Measuring gas emissions from livestock buildings: A review on uncertainty analysis and error sources. Biosyst. Eng..

[16] Lefcourt A.M. (2002). Some potential problems for measuring ammonia emissions from farm structures. Trans. ASABE.

[17] Heber A.J., Ni J.-Q., Lim T.T., Tao P.-C., Schmidt A.M., Koziel J.A., Beasley D.B., Hoff S.J., Nicolai R.E., Jacobson L.D., Zhang Y. (2006). Quality assured measurements of animal building emissions: Gas concentrations. J. Air Waste Manage. Assoc..

[18] Hinz T., Linke S. (1998). A comprehensive experimental study of aerial pollutants in and emissions from livestock buildings. Part 2: Results. J. Agric. Eng. Res..

[19] Wheeler E., Casey K., Gates R.S., Xin H., Zajaczkowski J.L., Topper P.A., Liang Y., Pescatore A.J. (2006). Ammonia emissions from twelve U.S.A. broiler chicken houses. Trans. ASAE.

[20] Topper P.A., Wheeler E.F., Zajaczkowski J.L., Gates R.S., Xin H., Liang Y., Casey K. (2008). Ammonia emissions from two empty broiler houses with built-up litter. Trans. ASABE.

[21] Chepete H.J., Xin H., Mendes L.B., Li H., Bailey T.B. (2012). Ammonia emission and performance of laying hens as affected by different dosages of Yucca schidigera in the diet. J. Appl. Poult. Res..

[22] Mendes L.B., Xin H., Li H. (2012). Ammonia emissions of pullets and laying hens as affected by stocking density and manure accumulation time. Trans. ASABE.

[23] Zhao Y., Pan Y., Rutherford J., Mitloehner F.M. (2012). Estimation of the interference in Multi-Gas measurements using infrared photoacoustic analyzers. Atmosphere.

[24] Hassouna M., Robin P., Charpiot A., Edouard N., Méda B. (2013). Infrared photoacoustic spectroscopy in animal houses: Effect of non-compensated interferences on ammonia, nitrous oxide and methane air concentrations. Biosyst. Eng..

[25] Nicoloso R.S., Bayer C., Denega G.L., de Oliveira P.A.V, Higarashi M.M., Corrêa J.C., Lopes L.S. (2013). Gas chromatography and photoacoustic spectroscopy for the assessment of soil greenhouse gases emissions. Ciência Rural St. Maria.

[26] Griffith D.W.T., Leuning R., Denmead O.T., Jamie I.M. (2002). Air–land exchanges of CO_2_, CH_4_ and N_2_O measured by FTIR spectrometry and micrometeorological techniques. Atmos. Environ..

[27] Grutter M. (2003). Multi-gas analysis of ambient air using FTIR spectroscopy over Mexico City. Atmosfera.

[28] Hashmonay R.A., Yost M.G., Mamane Y., Benayahu Y. (1999). Emission rate apportionment from fugitive sources using open-path FTIR and mathematical inversion. Atmos. Environ..

[29] Eklund B. (1999). Comparison of line– and point–source releases of tracer gases. Atmos. Environ..

[30] Piccot S.D., Masemore S.S., Ringler E.S., Srinivasan S., Kirchgessner D.A., Herget W.F. (1994). Validation of a method for estimating pollution emission rates from area sources using open-path FTIR spectroscopy and dispersion modeling techniques. Air Waste.

[31] Yasuda T., Yonemura S., Tani A. (2012). Comparison of the characteristics of small commercial NDIR CO_2_ sensor models and development of a portable CO_2_ measurement device. Sensors.

[32] Calvet S., Campelo J.C., Estellés F., Perles A., Mercado R., Serrano J.J. (2014). Suitability evaluation of multipoint simultaneous CO_2_ sampling wireless sensors for livestock buildings. Sensors.

[33] Jäger F., Wagner G., Meijer H.A.J., Kerstel E.R.T. (2005). Measuring δ^13^C of atmospheric air with non-dispersive infrared spectroscopy. Isotopes Environ. Health Stud..

[34] Frodl R., Tille T. (2006). A high-precision NDIR CO_2_ gas sensor for automotive applications. IEEE Sens. J..

[35] Park J., Cho H., Yi S. (2010). NDIR CO_2_ gas sensor with improved temperature compensation. Procedia Eng..

[36] Mendes L.B., Edouard N., Ogink N.W.M., van Dooren H.J.C., Tinôco I.F.F., Mosquera J. (2015). Spatial variability of mixing ratios of ammonia and tracer gases in a naturally ventilated dairy cow barn. Biosyst. Eng..

[37] Altman D.G., Bland J.M. (1983). Measurement in medicine: The analysis of method comparison studies. Statistician.

[38] Hodgkinson J., Smith R., Ho W.O., Saffell J.R., Tatam R.P. (2013). Non-dispersive infra-red (NDIR) measurement of carbon dioxide at 4.2 μm in a compact and optically efficient sensor. Sens. Actuators B Chem..

[39] SenseAir^®^ (2010). Technical Note: SenseAir® CO_2_ Sensors on Pressure Sensitivity, TN-025.

[40] Chagunda M.G.G., Yan T. (2011). Do methane measurements from a laser detector and an indirect open-circuit respiration calorimetric chamber agree sufficiently closely?. Anim. Feed Sci. Technol..

[41] Christensen J. (1990). Optical Filters and their Use with the Type 1302 & Type 1306 Photoacoustic Gas Monitors.

[42] USEPA (1998). Environmental Technology Verification Report: Photoacoustic Infrared Monitor, Innova AirTech Instruments Type 1312 Multi-Gas Monitor.

[43] Miles D.M., Rowe D.E., Owens P.R. (2008). Winter broiler litter gases and nitrogen compounds: Temporal and spatial trends. Atmos. Environ..

